# The complete mitochondrial genome of Patagonian moray cod, *Muraenolepis orangiensis* Vaillant, 1888 (Gadiformes, Muraenolepididae)

**DOI:** 10.1080/23802359.2020.1787275

**Published:** 2020-07-09

**Authors:** Eunkyung Choi, Seong Hee Park, Seung Jae Lee, Euna Jo, Jinmu Kim, Jeong-Hoon Kim, Jin-Hyoung Kim, Jong Seok Lim, Hyun Park

**Affiliations:** a Division of Biotechnology, College of Life Sciences and Biotechnology, Korea University, Seoul, Korea; b Department of Biological Science, Sookmyung Women's University, Seoul, Korea; c Unit of Research for Practical Application, Korea Polar Research Institute (KOPRI), Incheon, Korea; d Division of Polar Life Science, Korea Polar Research Institute, Incheon, Korea

**Keywords:** Mitochondria genome, *Muraenolepis orangiensis*, Patagonian moray cod, PacBio

## Abstract

The full-length mitochondrial genome of *Muraenolepis orangiensis* (Vaillant, 1888) was studied using PacBio platform and it is first report in a Muraenolepididae family. The circular form of mitochondria genome is 16,833 bp including 13 protein-coding genes, two rRNA, and 22 tRNA. Start codon of 13 protein-coding genes was only ATG but three types of stop codons (TAA, T(AA), and TAG) were detected. To evaluate evolutionary position of *M. orangiensis*, the phylogenetic tree with other 13 Antarctic fishes belonged to five families were showed that *M. orangiensis* is unique cluster as a Muraenolepididae family and this study would provide fundamental data to understand the evolutionary relationship of fishes founded in Antarctic area.

*Muraenolepis orangiensis* (Vaillant, 1888) is known as Patagonian moray cod but their biology and taxonomy are poorly studied. According to fishbase (https://www.fishbase.se/summary/7127), their depth distribution is 135 − 860 m and size is common to 20 cm. Also, distribution is Southwest Atlantic (Strait of Magellan (Ref. 27363), the Patagonian region of Argentina and Southern Ocean (Kerguelen, Heard, and Crozet islands). *M. orangiensis* is a Gadiform family which has eight species in two genera and five of these have been described in the last 10 years (Konstantinidis et al. 2016). In this study, we analyzed the first full-length mitochondrial genome of *M. orangiensis* using PacBio platform and phylogenetic tree was constructed to know the relationship with other fish families which are founded in Antarctic area.

The sample was collected from Southern ocean (65°05′S, 170°30′E on CCAMLR Subarea 88.1), Antarctica, and DNA was isolated using the conventional phenol-chloroform method. The specimen was deposited at the Earth Biocollection in the Division of Biotechnology, Korea University with accession number KAN0002030. For sequencing using PacBio, the 20 kb fragmentation step was conducted with Covaris G-tube (Covaris, Woburn, MA) and the SMRTbell library was constructed by using SMRTbell^™^ Template Prep Kit 1.0 (Pacific Biosciences, Menlo Park, CA) according to manufacturer’s protocol. The sequencing step was done using SMRT cells and sequencing kit in the Sequel sequencing platform (Pacific Biosciences, Menlo Park, CA). De novo assembly for mitochondria genome was performed by CANU assemble (Koren et al. 2017) after subread of PacBio for mitochondria genome assembly were filter out using 16 s *rRNA* and *COI* gene sequences. The assembled sequences were retrieved into MITOS (Bernt et al. 2013) web service for mitochondrial genome annotation.

The complete mitochondrial genome size of *M. orangiensis* (GenBank Number: MT192937) was 16,833 bp including 13 protein-coding genes, two rRNAs, and 22 tRNAs. Start codon of 13 protein-coding genes was only ATG but three types of stop codons (TAA, T(AA), and TAG) were detected. Seven protein-coding genes (*ND1*, *ATP8*, *ATP6*, *CO3*, *ND3*, *ND4L*, and *ND5*) had TAA as a stop codon and TAG stop codon was for three protein-coding genes (*ND2*, *CO1*, and *ND6*). T(AA) stop codon was for *ND4*, *Cytb*, and *CO2* protein-coding genes.

A phylogenetic tree (Figure 1) was conducted to evaluate evolutionary position of *M. orangiensis*. We used protein sequence of 13 protein-coding genes of known complete mitochondria genomes of 13 Antarctic fishes and these Antarctic fishes are belong to five families, *Channichthyidae*, *Artedidraconidae*, *Nototheniidae*, *Eleginopidae*, and *Gadidae*. MEGA X software was conducted and maximum-likelihood method and JTT matrix-based model were used (Jones et al. 1992; Kumar et al. 2018). The phylogenetic tree showed *M. orangiensis* is different with Gadidae family and it is unique cluster as a Muraenolepididae family. This study was performed first time to better understand *M. orangiensis* in evolutionary position with other fishes living in Antarctic area.

**Figure 1.**
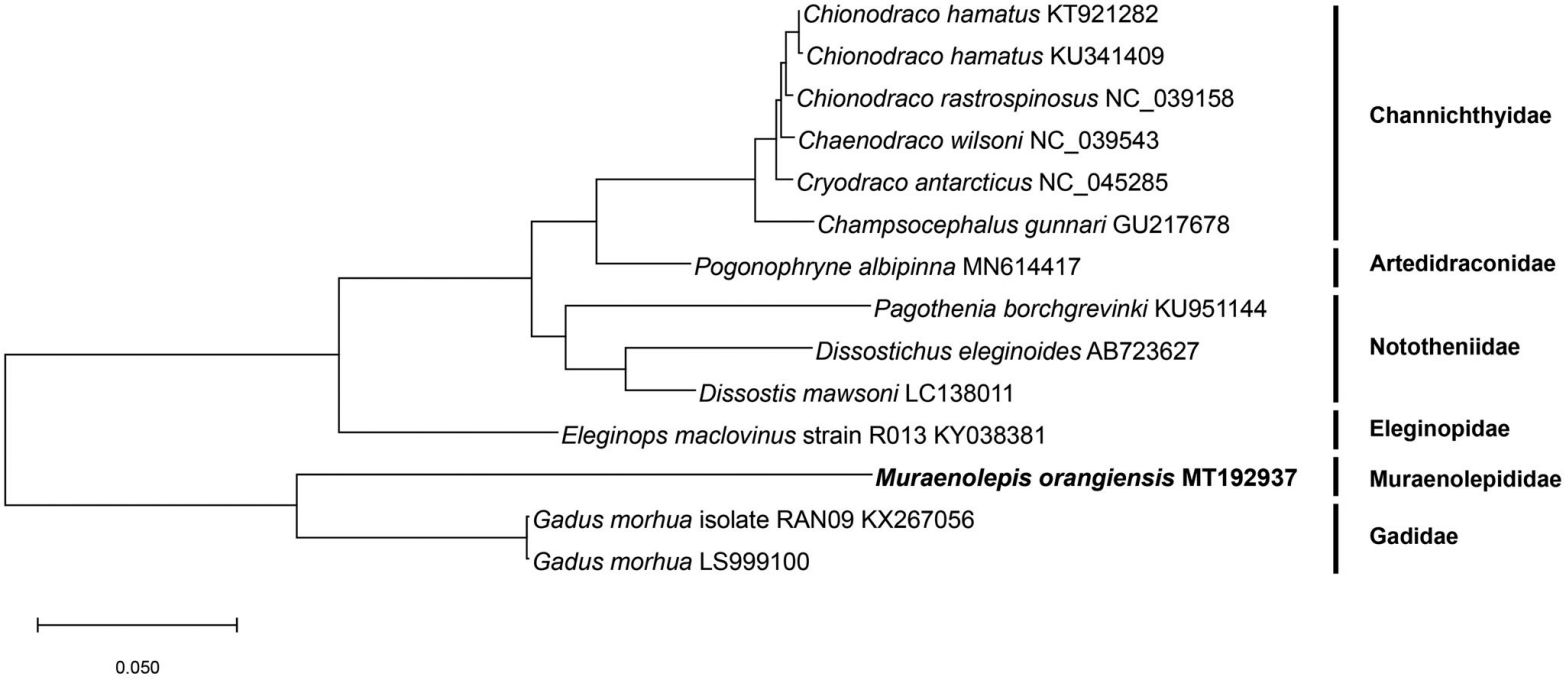
Phylogenetic tree was constructed with protein sequences of 13 protein-coding genes of 14 Antarctic fishes using. The maximum-likelihood method and JTT matrix-based model. Scientific name and GenBank number are indicated for each species.

## Data Availability

The data that support the findings of this study are openly available in NCBI under the accession MT192937 (https://www.ncbi.nlm.nih.gov/nuccore/MT192937.1).
